# Experiences from tsunami relief activity: implications for medical education

**Published:** 2012-09-30

**Authors:** Sudharsanam Manni Balasubramaniam, Yogesh Mohan, Gautam Roy

**Affiliations:** Department of Preventive & Social Medicine, Jawaharlal Institute of Postgraduate Medical Education & Research (JIPMER), Pondicherry, India

## Abstract

A tsunami struck the coast of Tamilnadu and Pondicherry on 26 December 2004. Jawaharlal Institute of Postgraduate Medical Education & Research, (JIPMER) in Pondicherry played a vital role in providing medical relief. The experiences from the relief activities revealed areas of deficiency in medical education in regards to disaster preparedness. A qualitative study using focus group discussion was employed to find the lacunae in skills in managing medical relief measures. Many skills were identified; the most important of which was addressing the psychological impact of the tsunami on the victims. Limited coordination and leadership skills were also identified. It is recommended that activity-based learning can be included in the curriculum to improve these skills.

## Introduction

On the morning of 26 December 2004, a tsunami struck the coastal areas of Pondicherry and Tamilnadu in south India, causing large-scale destruction of property, deaths, and human suffering. Relief work was started immediately, and JIPMER played an important role in providing medical relief. All resident doctors and faculty of the Department of Preventive and Social Medicine (PSM), along with residents and faculty from other departments, participated. Though disaster management is taught in the curriculum in PSM, our observation of the situation in the field was different. Post-tsunami health problems were minor as many of the affected people had abrasions or minor injuries. Rather, these people needed mental health support. During the relief work in the field we observed and realized the lacunae among the resident doctors regarding the skills required to address disaster as a health professional and manager. These limitations have also been identified in Sri Lanka by medical professionals.^1^ Although medical professionals are included in disaster preparedness, their preparation is limited and tends to focus mainly on medical management, to the exclusion of social support.^2^,^3^ Based on our experience providing support to people stricken by a tsunami, we have identified limitations in skills that must be included in a medical curriculum to prepare medical personnel when faced with disaster management.

## Methods

This study was of a qualitative, one time snapshot design, through a focus group discussion held among resident doctors of PSM and other departments who were part of the teams managing the post-tsunami medical relief. A total of ten resident doctors who were active participants in the tsunami relief were purposively sampled and included in the discussion. The questions were guided using an informal schedule. Notes were recorded, and an iterative process of analysis was used to identify themes.

## Results

A total of four themes emerged.

### Skills in supporting mental health

The majority of people impacted by the tsunami had lost their house, property or kith and kin. As a result, they were in need of mental health support. Counseling skills were clearly necessary in mitigating the suffering of the survivors and helping them cope with the disaster. This theme is exemplified in the following comment:

We did not know how to handle so many psychologically affected people in large numbers…

### Coordination skills

District health authorities asked health teams to report to them in person every morning. This required considerable time and use of resources to travel. In addition, several teams from various states and Non-Governmental Organizations (NGOs) were involved in relief work, without coordinating with health authorities. Hence there was duplication of work.

We had to travel 50 kilometers in a few days just to find the next site of our camp. It was just a waste of time and petrol.

### Leadership skills

Leadership skills were also lacking. Managing relief teams, motivating workers, and interacting with diverse groups of people or different sections of government and NGOs are critical when dealing with crisis situations. In addition, interpersonal and communication skills including sharing, caring and empathizing with the affected people were also identified. Most residents reported being emotionally distanced from their patients and their feelings when working in a large hospital like JIPMER. In a disaster situation, however, closeness and empathy were important.

I could see that many of my colleagues were treating them just like a patient. They could not understand their emotions and feelings - which actually they wanted us to know.

### Lack of interest in minor illnesses

Since most of the resident doctors were working in a hospital setting during their residency, they had become accustomed to managing “big” or complicated cases - minor illnesses were regarded as less important. Although they treated and reassured the tsunami-stricken patients, they were disappointed by the limited medical challenges. For example, during the relief work some resident doctors commented,

Let’s go back; what are we doing here…?

We’re not doing anything….

I have to go back…my exams are coming up.

## Discussion

Each disaster is unique, depending on the geographic region, the socio-economic status of the population, and the nature and ferocity of the disaster. The tsunami that struck the Indian subcontinent was an unusual event in the region and caused a great loss to life and property. Though medical teams effectively managed various medical contingencies, there was a general lack of skill demonstrated in the management of the psychological impact of the disaster. These results suggest the need for teaching residents about the psychological consequences of disasters and means of coping.^4^ Communication and counseling skills are seldom taught in medical schools, and these are critical in disaster situations. Though many of these skills cannot be readily transferred as knowledge in a lecture or seminar, they can be highlighted through role plays, disaster drills, and mimicking field situations. It is necessary that such activity-based skill learning is included in the medical curriculum so that health professionals are better equipped to handle the impact that disasters have on the survivors.

### Conclusions

Counseling, coordination, and leadership skills are considered critical according to medical residents who were involved in providing medical support in these areas. They also felt that they lacked the skills to handle the psychological impact of the disaster on the affected population.
